# Tablets before liquids? Rethinking paediatric prescribing in primary care

**DOI:** 10.3399/BJGP.2025.0652

**Published:** 2026-03-10

**Authors:** Ahmed Elkhazragy, Alleh Jonroy, Rebecca Elizabeth Payne

**Affiliations:** 1 Egypt-Air Hospital, Cairo, Egypt; 2 Royal Free Foundation Trust, London, UK; 3 North Wales Medical School, Bangor University, Gwynedd, UK

## Introduction

Liquid medicines are commonly prescribed for children, yet they are often the least practical option. Syrups are more expensive than tablets and can be difficult for families to store, transport, or measure accurately. Many liquids contain sugars or excipients that carry their own harms. In contrast, tablets and capsule formulations provide accurate dosing, longer shelf life, and lower cost — and many children can swallow them far earlier than commonly assumed. It may be time to ask: should tablets come before liquids in paediatric prescribing?

## The case for change

Although liquid formulations are often considered the default for children, they present multiple practical and clinical challenges. They are typically more expensive than solid oral dosage forms,^1^ and supply shortages are common, often forcing parents to visit several pharmacies or requiring prescribers to issue alternative preparations to fit local availability.^2^ Administration during the school day is often impractical, and longer treatment courses may necessitate frequent pharmacy visits or reconstitution at home. Many liquids require refrigeration, which is difficult for families in asylum accommodation or in areas with unreliable power.^3^ In addition to these practical barriers, liquid preparations raise concerns about dosing accuracy and stability and wastage.^4,5^ Liquid medicines are also vulnerable to administration errors at home, where families may use teaspoons or cups rather than oral syringes, and, despite parents’ best efforts, taste issues often compromise adherence. Excipients present further risks, as many paediatric syrups contain sugars, sorbitol, or other additives that may cause dental or gastrointestinal harms.^6^

## But what are the alternatives?

Despite the challenges with liquid medication, it is rare for GPs to prescribe alternatives for young children. But is this justified? Many of the problems of liquid medication are overcome by other formulations. Although rectal administration is unlikely to be popular in the UK (unlike in other European countries), tablets and capsules provide validated strengths with long shelf-lives. Similarly, dispersible tablets and powder sachets, combine reliable dosing and good shelf stability with sugar-free or lower-excipient formulations.

## Are tablets suitable for children?

Contrary to longstanding assumptions, many preschool and school-age children can learn to safely swallow tablets after brief, structured coaching. Programmes such as KidzMed report rapid acquisition of this skill in children aged ≈4 years and older,^7^ supporting wider use of solid oral forms for this age group.^8^ Box 1 provides links to resources that can be shared with families, many of whom find tablets considerably easier to manage. Box 2 provides a parent’s experience. Even when a child cannot take an intact tablet, crushing, splitting, or opening tablets or capsules may be appropriate.

Box 1. **Resources for families and healthcare professionals**

**Resources to share with families**
Videos on taking tablets and other medication: Medicines for Children, https://www.medicinesforchildren.org.uk/advice-guides/giving-medicines/
Information on preparing antibiotic powders: Alder Hey,  https://www.alderhey.nhs.uk/conditions/patient-information-leaflets/how-to-prepare-reconstitute-an-antibiotic-powder-for-oral-solution-m34/
**Resources for healthcare professionals**
E-learning on children and tablets: KidzMed, https://learninghub.nhs.uk/catalogue/KidzMed
Advice on crushing tablets: Specialist Pharmacy Service, https://www.sps.nhs.uk/articles/checking-if-tablets-can-be-crushed-or-capsules-opened/

Box 2. **A parent’s story**

My 6-year-old with cystic fibrosis has been swallowing tablets since he was not quite 3, and it has been so much more convenient. Liquids were a constant hassle: Creon Micro came as tiny granules that went everywhere, antibiotics had to be kept in the fridge, and a 2-week course meant the second week needed reconstituting either at the pharmacy or at home. Bottles were bulky to travel with, prone to leaking, and always needed syringes or spoons for measuring — fiddly, messy, and often inaccurate.
With tablets, everything is easier and more discreet. I can slip them into my pocket and give them at parties without anyone noticing. The main frustration now is when a GP or pharmacy, thinking they are being helpful because my child is small, switches prescriptions back to liquid. That creates more work for us in chasing, ordering, and collecting medicines.
Interestingly, we discovered tablet-swallowing almost by accident: we had been opening capsules and he simply swallowed the empty shell. Later we tried yoghurt, spreads, and Tic Tacs, but he needed very little practice. Now I’m about to start with my 3-year-old. This morning his big brother proudly showed him how to do it — we’ll see how it goes over the next week!

## So should GPs change their prescribing practices?

It might be time to consider a pragmatic tiered approach:

1. Neonates and infants (including some toddlers): licensed liquid suspensions remain the primary option because of swallowing limitations and age-specific dosing needs.
2. Children ≈4 years and older who can swallow or can be coached quickly: tablets can be an excellent option. This is particularly so when small tablets or minitablets or licensed dispersible tablets are available, maximising dosing accuracy and minimising excipient exposure. Box 1 contains resources that can be shared with families to support training in taking tablets.
3. Non-swallowers or where tablet strengths do not match weight bands: in some cases premeasured powder sachets can be found. Clinicians should make sure reconstitution kits and instructions are provided.
4. Complex needs (developmental disability, swallowing disorder, severe allergies): a discussion with community pharmacy or the paediatricians may be useful.

Many practices have embedded pharmacists who may be able to provide coaching, check compatibility of crushed or dispersed products, and advise on reconstitution. Similarly, community pharmacists can provide advice directly to parents when they collect medication.

## Practical considerations

### Can parents/carers safely reconstitute powdered antibiotics?

Yes. However, several conditions should be met and are described in Box 3.

Box 3. **Parental reconstitution of powdered antibiotics**

**Required product and kit elements**

- Pre-measured, sealed sachets or labelled dry bottles specifying drug, strength, batch, and expiry.
- A simple reconstitution kit supplied with each powder dispensing: a calibrated oral syringe (5–10 mL) or marked cup matching the required volume, a clean mixing container or the original bottle, and a cap or seal to allow vigorous shaking.
- Clear, multimodal instructions: a one-page pictogram checklist, a QR-linked 30–60 second demonstration video (Medicines for Children/KidzMed style), and/or a printed label showing ‘prepared on/discard after’ (typically 7–14 days as per manufacturer guidance).
- A helpline number for pharmacy/clinician queries during first administration.

**Process and safety steps**

- Clinician confirms allergy status and suitability during the consultation and records formulation choice. Parents/carers should be counselled on hand hygiene, boiling/cooling water where required by local instruction, measuring exact water volume, emptying the sachet fully, shaking until dissolved, labelling and storing as directed, and discarding after the stated period.
- Reinforce taste-masking options (small spoonful of yoghurt or jam) and use of an oral syringe to deliver into the cheek pocket for younger children.
- Advise parents/carers that reconstituted preparations are for single-patient use, to be kept out of reach of children, and to seek advice if colour, odour, or consistency is abnormal.

### Is opening capsules and crushing tablets a sensible alternative to liquids?

Guidance from the Specialist Pharmacy Service and tertiary centres such as Great Ormond Street Hospital highlights that intact tablets and capsules are the preferred choice.^9^ Whenever children can safely take solid oral dosage forms, these should be prescribed. When this is not possible, and there is no liquid option available, crushing tablets or opening capsules may be considered. However, this is off-label, and so should only be done where there are no suitable alternatives. Prescribers should bear in mind that this alters pharmacokinetics and should not be considered for modified-release or enteric-coated products where it can cause therapeutic failure or toxicity.^10,11^ When considering this, GPs should check the Specialist Pharmacy Service website and follow their protocols. If crushing is necessary and permitted by the Summary of Product Characteristics (SmPC), clear advice and clear, step-by-step instructions must be provided to parents. Clinicians should emphasise the need for immediate administration, and provide allergy warnings and personal protective equipment guidance as appropriate.^9,12,13^

## Conclusion and recommendation

For many children, tablets are not only possible but also preferable. They reduce cost, improve dosing accuracy, avoid unnecessary excipients, and are easier for families to manage than bulky syrups. With simple coaching, even preschoolers can learn to swallow small tablets, and dispersible or powder forms provide further options. As crushing tablets or opening capsules are off-label and can carry risks, this should only be undertaken as a final resort, for example, during serious supply shortages or for specialist medication only available in this form. Liquids will remain necessary for infants and those with complex needs, but they need not be the default. By prescribing tablets rather than liquids, GPs can support safer, simpler, and more sustainable prescribing for children.
